# Eligibility for Anti‐Amyloid‐β Monoclonal Antibodies in Patients With Primary Progressive Aphasia due to Alzheimer's Disease in Japan

**DOI:** 10.1111/psyg.70152

**Published:** 2026-03-06

**Authors:** Shoya Inagawa, Yuta Inagawa, Akito Tsugawa, Naoto Takenoshita, Kazuhiro Saito, Kenji Ishii, Kensaku Kasuga, Takeshi Ikeuchi, Soichiro Shimizu

**Affiliations:** ^1^ Department of Geriatric Medicine Tokyo Medical University Tokyo Japan; ^2^ Department of Radiology Tokyo Medical University Tokyo Japan; ^3^ Tokyo Metropolitan Geriatric Hospital and Institute of Gerontology Tokyo Japan; ^4^ Brain Research Institute, Niigata University Niigata Japan; ^5^ Department of Diagnostic Innovation Science National Center for Geriatrics and Gerontology Aichi Japan

**Keywords:** Alzheimer's disease, Logopenic variant, primary progressive aphasia, single‐photon emission computed tomography, standard language test of aphasia

## Abstract

**Background:**

Primary progressive aphasia (PPA) is a clinical syndrome characterized by progressive language impairment. The logopenic variant (lvPPA) is frequently associated with Alzheimer's disease (AD) pathology. With the approval of anti‐amyloid‐β monoclonal antibodies, such as lecanemab and donanemab, for the treatment of AD, accurately differentiating lvPPA due to AD (lvAD) from the other PPA variants has become highly important.

**Methods:**

Thirteen patients with PPA who underwent cognitive testing, including the Standard Language Test of Aphasia (SLTA), brain magnetic resonance imaging, single‐photon emission computed tomography (SPECT), cerebrospinal fluid (CSF) biomarker analysis, and amyloid positron emission tomography (PET) imaging were enrolled in this study. Patients were classified into the lvPPA, semantic variant PPA (svPPA), and nonfluent/agrammatic variant PPA (nfaPPA) groups based on the results of clinical and neuropsychological assessments. We determined the proportion of PPA patients in each group who met the optimal use guidelines for anti‐amyloid monoclonal antibody therapy.

**Results:**

Six (86%) of the 7 lvPPA patients were amyloid positive (A+), and 5 (71%) were eligible for lecanemab or donanemab. In most lvPPA patients, SPECT displayed an AD‐typical pattern of hypoperfusion in the bilateral temporoparietal regions, posterior cingulate gyrus, and precuneus. One lvPPA patient was negative for AD biomarkers and demonstrated an atypical perfusion pattern for AD, and was subsequently rediagnosed as having frontotemporal lobar degeneration.

**Conclusions:**

These results suggest that SPECT may play a supportive role in differentiating lvAD from PPA. In the current clinical era of anti–amyloid‐β monoclonal antibody therapy, accurate identification of lvAD among patients with language‐predominant symptoms is increasingly important. Larger studies should be performed in the near future to validate these findings.

## 1 Introduction

Primary progressive aphasia (PPA) is a clinical syndrome including neurodegenerative diseases characterised by predominant speech and language impairment. Since Mesulam first described slowly progressive aphasia in 1982 [1], numerous cases of PPA have been reported. In 2011, Gorno‐Tempini proposed diagnostic criteria and a classification system for PPA [2], which has gained widespread international acceptance. The diagnosis of PPA is based on the following three inclusion criteria: (1) the most prominent clinical feature is difficulty with language, (2) these deficits are the principal cause of impaired daily living activities, and (3) aphasia should be the most prominent deficit at symptom onset and for the initial phases of the disease. PPA is classified into three subtypes, namely, the logopenic variant (lvPPA), the semantic variant (svPPA), and the nonfluent/agrammatic variant (nfaPPA). Each subtype is characterised by distinct language impairments and has its own diagnostic criteria. Additionally, each subtype is associated with different underlying neuropathologies. Specifically, lvPPA is most often associated with Alzheimer's disease (AD) pathology, svPPA with frontotemporal lobar degeneration with TDP‐43 pathology, and nfaPPA with frontotemporal lobar degeneration with tau pathology. Notably, approximately 80% of lvPPA cases are associated with AD pathology, representing a very high proportion [3].

The anti‐amyloid‐β monoclonal antibodies lecanemab [4] and donanemab [5] were recently approved as disease‐modifying therapeutic agents for AD. It is important to note that these antibodies are indicated not only for typical amnesic AD but also for lvPPA due to AD [6] Therefore, it is important to accurately differentiate lvPPA due to AD from PPA.

The PPA subtypes were classified according to the diagnostic criteria proposed by Gorno‐Tempini et al. [2]. through a combination of detailed clinical assessment of speech and language symptoms by experienced clinicians and neuroimaging findings. In clinical practice, clinical assessment tools including the Western Aphasia Battery [7] or its Japanese version [8] are often used to evaluate aphasia. In addition, in Japan, the Standard Language Test of Aphasia (SLTA) [9], which is a reliable and validated tool for assessing aphasia symptoms, is commonly used to classify PPA subtypes [9]. However, even upon performing detailed language assessments including the SLTA, patients with different PPA subtypes often present with similar and overlapping symptoms, making the identification and classification of PPA challenging. For example, the core features of lvPPA, such as impaired single‐word retrieval in spontaneous speech, and the naming or impaired repetition of sentences and phrases, are not uncommon in patients with aphasia, and the language‐associated symptoms of lvPPA lack distinctive and definitive features. Therefore, it is sometimes difficult to classify PPA into subtypes using aphasia assessments, and single‐photon emission computed tomography (SPECT) is sometimes used as an adjunct for the classification of PPA. It is also known that each PPA subtype tends to lead to reduced cerebral blood flow (rCBF) in different brain areas. The regions of rCBF are the angular gyrus and supramarginal gyrus in lvPPA, the left anterior temporal lobe in svPPA, and the precentral gyrus and inferior frontal gyrus in nfaPPA [10, 11]. However, there are few reports to date on the systematic application of SPECT findings to classify PPA subtypes, and even fewer reports that address the importance of PPA subtype classification in the present era of anti‐Aβ antibody therapy.

As mentioned above, in the present era of anti‐amyloid‐β antibody therapy, it is important to accurately differentiate lvPPA due to AD from PPA. Therefore, in this study, we aimed to differentiate patients with lvPPA due to AD who are eligible for anti‐amyloid‐β antibody therapy from patients along the PPA continuum in our medical practice. To our knowledge, this is the first clinical study in Japan, in the era of anti‐amyloid‐β antibody therapy that concurrently evaluates language function and functional brain imaging in PPA patients.

## 2 Methods

### 2.1 Patients

Thirteen patients with predominant speech and language impairment who presented to the Memory Disorder Clinic at the Department of Geriatric Medicine, Tokyo Medical University, between December 2019 and September 2023 were enrolled in this study. All patients underwent cognitive assessment tests, including SLTA, which were administered by a clinical psychologist with more than 10 years of professional experience. Based on the diagnostic criteria proposed by Gorno‐Tempini et al. [2], 3 neurologists (i.e., S.S., N.T., and Y.I.) made the diagnosis and classified each patient into the lvPPA, svPPA, and nfaPPA groups. In addition, they underwent general physical examinations, blood tests to exclude other potential causes of dementia, head magnetic resonance imaging (MRI) and cerebral blood flow analysis by SPECT to establish a clinical diagnosis. None of the patients had any history of cerebrovascular disease or infarction in the region of the intracranial lesions on brain MRI. To confirm AD pathology in the patients, cerebrospinal fluid (CSF) was collected by lumbar puncture, and CSF amyloid‐β42/40 (Aβ42/40) ratio and phosphorylated tau at threonine 181 (pTau181) were analysed at the Brain Research Institute of Niigata University [12, 13]. According to our institutional criteria, a decreased CSF Aβ42/40 ratio (< 0.068) and an increased CSF pTau181 level (>29.0 pg/mL) were considered indicative of AD pathology. The patients also underwent amyloid positron emission tomography (PET) at Tokyo Metropolitan Geriatric Hospital and Institute of Gerontology, and Tokyo Medical University Hospital. According to the ATN classification system [14], patients were designated as A+ if they showed decreased CSF Aβ42/40 (< 0.068) or positive amyloid PET findings, and as T+ if they showed increased CSF pTau181 (>29.0 pg/mL). Various clinical characteristics were compared among the lvPPA, svPPA, and nfaPPA groups. In this manuscript, lvPPA due to AD is referred to as “lvAD”. Among the lvAD patients, we also reported the proportion who met the optimal use guidelines (OUG) [15, 16] for anti‐amyloid‐β monoclonal antibody therapy. Although the Mini‐Mental State Examination (MMSE) eligibility thresholds differ slightly between lecanemab and donanemab, we uniformly applied an MMSE range of 20–30 points for the purpose of this analysis. Details of the eligibility criteria are provided in the OUG (see website). The protocol of this study was approved by the Ethics Committee of Tokyo Medical University (study approval no.: T2023‐0214). Informed consent was obtained from all study participants (either the patients themselves or their closest relative) before enrollment in the study, following a detailed explanation of the aim of the study.

### 2.2 Cognitive Assessment Test

As part of the cognitive assessment test, the MMSE [17], the Japanese version of the Montreal Cognitive Assessment (MoCA‐J) [18], AD Assessment Scale‐Cognition (ADAS‐Cog) [19], Frontal Assessment Battery (FAB) [20], and SLTA were performed. The SLTA, developed by the Japan Society for Higher Brain Dysfunction in 1975, is a widely used and validated tool for the diagnosis of aphasia and the planning of patient treatment in clinical practice. It consists of 26 subtests covering the following 5 domains: hearing, speaking, reading, writing, and calculation. Each subtest is scored on a 6‐point scale, and the resulting profile visually represents both the severity and type of aphasia. SLTA provides a detailed understanding of aphasic symptoms and their progression, making it a valuable tool in rehabilitation settings.

### 2.3 Image Analysis

#### 2.3.1 SPECT

All patients underwent imaging using a triple‐head rotating gamma camera (e.cam, Siemens) with a fan‐beam collimator at a spatial resolution of 6.8‐mm full width at half maximum. Imaging was commenced at 15 min after intravenous injection of 222 MBq of N‐isopropyl‐p‐[^123^I] iodoamphetamine. In practice, the full width at half maximum would be higher (approximately 10–12 mm) when scanning patients because of the increase in source to collimator distances together with the effects of scattering within the patients, both of which degrade spatial resolution. Prior to the injection, the patients sat in a room with a quiet and relaxed atmosphere, with their eyes open for 10 min. After the injection, the patients lay down with their eyes closed during the imaging. SPECT images were acquired in 24 steps (72 projections), each of which collected counts for 40 s. Reconstruction of the images was performed by filtered back‐projection using Butterworth and Ramp filters (order: 8; cutoff: 0.40/cm) with attenuation correction (Chang method, 0.09/cm). The matrix size and slice thickness of the SPECT images were 128 × 128 mm and 3.3 mm, respectively.

Three‐dimensional stereotactic surface projections (3D‐SSP) created using Neurological Statistical Image Analysis Software developed by Minoshima et al. were applied to the ^123^I‐IMP SPECT images to generate 3D cerebral blood flow (CBF) images and Z‐score maps [21]. Region of interest (ROI) analysis was used to measure rCBF in the Brodmann Areas (BAs) associated with each subtype [10, 11] using stereotaxic extraction estimation (version 2.1) software (Nihon Mediphysics, Tokyo, Japan) [22]. The normalised brain activity of each patient was compared with that of 28 normal controls using pixel‐by‐pixel Z‐score analysis, defined as (individual value − normal mean) / normal Standard Deviation. The areas responsible for lvPPA, svPPA, and nfaPPA were designated as BA39 + 40 + 22, BA20 + 21 + 38, and BA44 + 45, respectively, in accordance with a previous study [10, 11]. The average pixel values of each ROI was normalised to the cerebellum and used for analysis.

#### 2.3.2 Amyloid PET

For imaging with Pittsburgh compound B (PiB), a total of 555 MBq of [^11^C] PiB was administered intravenously. Patients underwent either a 70‐min dynamic scan or a 20‐min static scan using a PET scanner, either 40 or 50 min after administration of the radiotracer. For imaging with flutemetamol, a total of 185 MBq of [^18^F] flutemetamol was administered intravenously. Patients underwent either a 70‐min dynamic scan or a 20‐min static scan using a PET scanner, either 40 or 50 min after administration of the radiotracer. For imaging, Discovery PET/CT 710 (GE Healthcare, Chicago, IL, USA) and Headtome V (Shimadzu Corporation, Kyoto, Japan) were used. Amyloid PET is a diagnostic test used to detect and quantify Aβ in the brain. In this study, the Centiloid method was used, which is a quantitative assessment of amyloid accumulation, with an average of 0 for young normal AD‐negative individuals and 100 for mild or moderate typical AD patients [23, 24]. Amyloid positivity was defined based on previously reported Centiloid cut‐off values (approximately 25–35), which have been shown to correlate closely with core AD biomarkers [25].

#### 2.3.3 Statistical Analysis

Analysis of Variance (ANOVA), the χ^2^ test, the Kruskal‐Wallis test, and the Steel‐Dwass test were used for the analysis of patient characteristics and CBF by SPECT. All values obtained are expressed as the mean ± SD. Data were statistically analysed using IBM SPSS statistics version 25 software (Chicago, IL, USA). A *p*‐value of less than 0.05 was considered to indicate a statistically significant difference between groups.

## 3 Results

**FIGURE 1:**
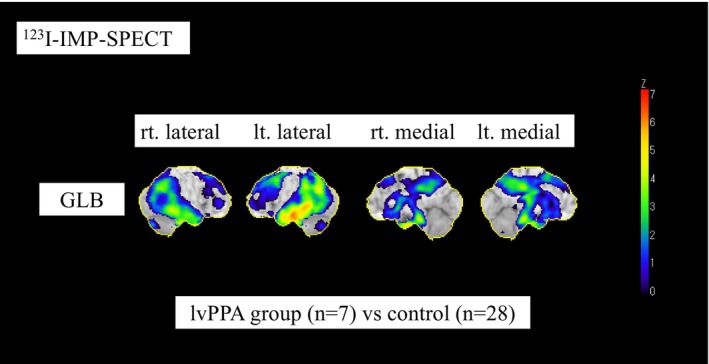
Reduction in cerebral blood flow measured by SPECT in the lvPPA group compared with the control group. A reduction in cerebral blood flow measured by SPECT in the lvPPA group compared with the control group is shown using 3D‐SSP. The SPECT results of patients in the lvPPA group displayed typical AD patterns.

Table 1 shows the characteristics of the patients in the aphasia groups. The lvPPA group consisted of 3 men and 4 women (age: 64.3 ± 8.9 years), the svPPA group consisted of 3 men and 1 woman (age: 67.0 ± 12.3 years), and the nfaPPA group consisted of 2 women (age: 63.0 ± 8.5 years). The 3 neurologists were in agreement regarding the classification of all patients. There were no significant differences in age, sex, and scores of MMSE, MoCA‐J, ADAS‐Cog, and FAB among the 3 groups. None of the patients were excluded based on MRI findings specified in the OUG, such as cerebral microhemorrhages or edema. Six (86%) of the 7 lvPPA patients were amyloid positive (A+), and 5 (71%) of the 7 patients would be eligible for lecanemab or donanemab. In the svPPA group, there were no amyloid‐positive (A+) patients who met the eligibility criteria for anti‐amyloid‐β monoclonal antibody therapy. In the nfaPPA group, 1 of the 2 patients showed a decreased CSF Aβ42/40 ratio of 0.067 (A+), whereas amyloid PET was negative with a Centiloid value of approximately 10 (A−). None of the nfaPPA patients met the eligibility criteria for antibody therapy. Figure 1 shows a rCBF measured by SPECT in the lvPPA group compared with the control group. The SPECT data show reduced cerebral blood flow in the bilateral temporal parietal lobes and the posterior cingulate gyrus to the precuneus, particularly in the left superior temporal gyrus and posterior part of the middle temporal gyrus.

**TABLE 1:** Characteristics of patients in the three aphasia groups.

	lvPPA	svPPA	nfaPPA	
	*n* = 7 (54%)	*n* = 4 (31%)	*n* = 2 (15%)	*p*
Age (years)	64.3 ± 8.9	67.0 ± 12.3	63.0 ± 8.5	0.96^a^
Sex (male/female)	3/4	3/1	0/2	0.38^b^
MMSE score	23.4 ± 4.6	22.3 ± 5.7	25.0 ± 7.1	0.79^c^
MoCA‐J score	17.1 ± 7.0	16.5 ± 3.5	19.0 ± 5.7	0.72^c^
ADAS score	15.8 ± 6.8	23.2 ± 12.9	10.5 ± 4.9	0.31^c^
FAB score	12.3 ± 2.6	10.5 ± 2.5	11.0 ± 2.8	0.62^c^
CSF Aβ42/40 positive	6 (86%)	0 (0%)	1 (50%)	—
Amyloid PET positive	6 (86%)	0 (0%)	0 (0%)	—
CSF ptau181 positive	7 (100%)	4 (100%)	0 (0%)	—
Treatment with anti‐amyloid‐β antibodies	5 (71%)	0 (0%)	0 (0%)	—

Abbreviations: ADAS‐cog, AD Assessment Scale‐Cognition; CSF, cerebrospinal fluid, FAB, Frontal Assessment Battery, MMSE, Mini‐Mental State Examination; MoCA‐J, Japanese version of the Montreal Cognitive Assessment; PET, Positron Emission Tomography.

^a^ ANOVA.

^b^ *χ* ^2^ test.

^c^ Kruskal‐Wallis test.

**FIGURE 2:**
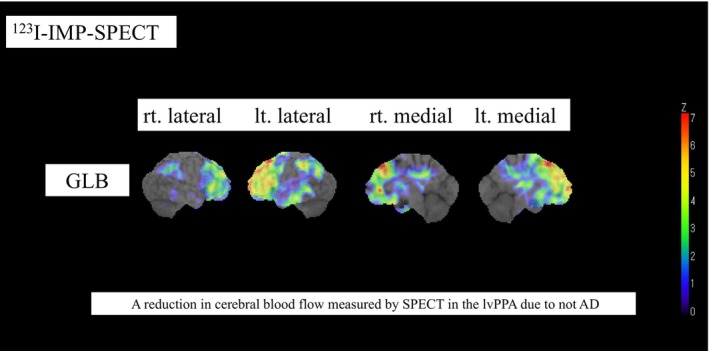
Reduction in cerebral blood flow (rCBF) measured by SPECT in the patients with lvPPA not due to AD. A reduction in cerebral blood flow (rCBF) measured by SPECT in patients with lvPPA not due to AD is shown using 3D‐SSP. The SPECT results displayed rCBF in the frontal lobes, which is an atypical pattern for AD.

Figure 2 shows a rCBF measured by SPECT of the 1 lvPPA patient without AD pathology (A– case). The SPECT data showed rCBF in the frontal lobes, which is an atypical pattern for AD.

**FIGURE 3:**
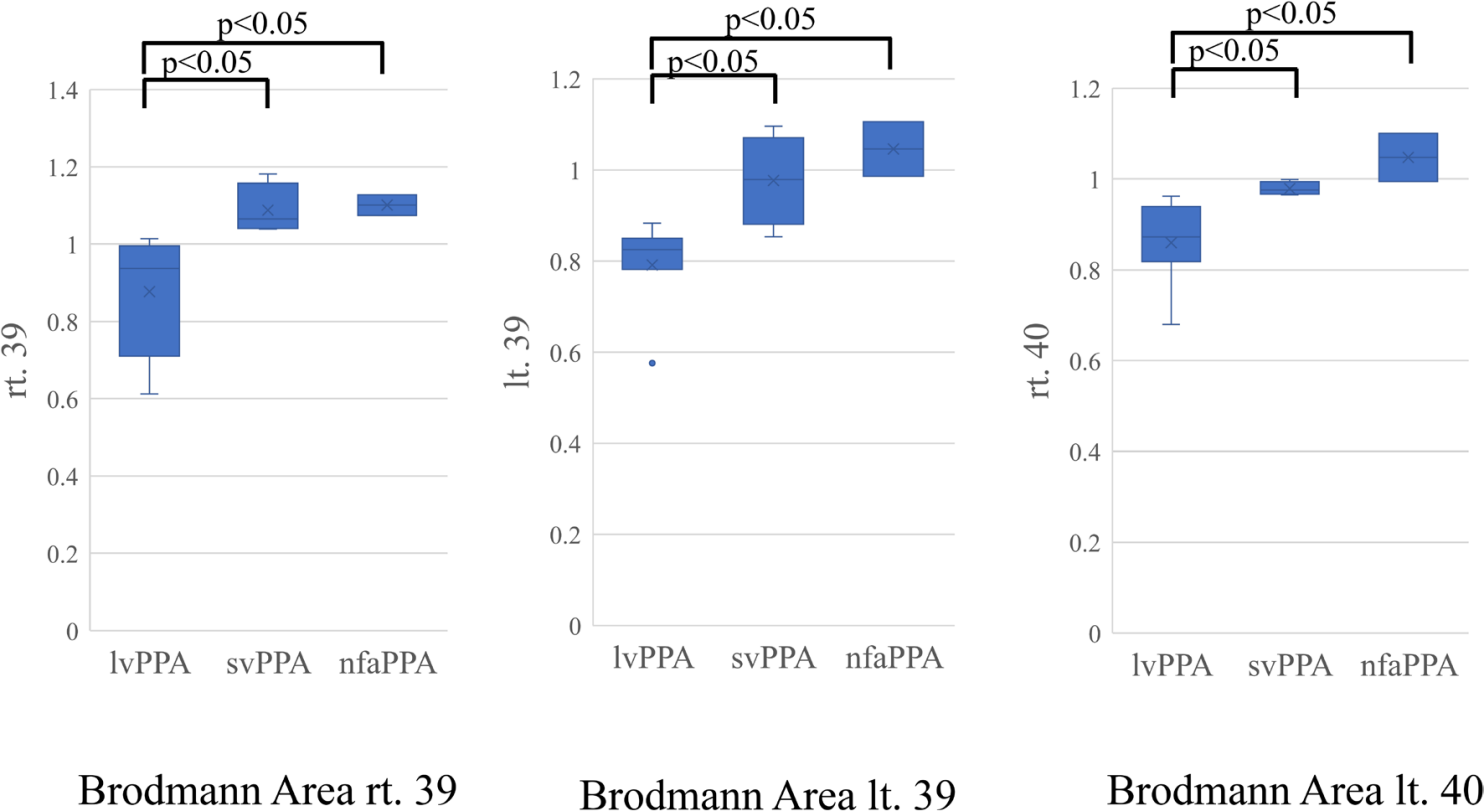
Comparisons of the reduction in cerebral blood flow measured by SPECT in BAs rt. 39, lt. 39, and rt. 40 among the lvPPA, svPPA, and nfaPPA groups. Comparisons of the reduction in cerebral blood flow measured by SPECT in BAs rt. 39, lt. 39, and rt. 40 among patients in the lvPPA, svPPA, and nfaPPA groups were performed using the Steel‐Dwass test. Significant differences were observed in BAs rt. 39, lt. 39, and rt. 40 between the lvPPA group and the svPPA group, as well as between the lvPPA group and the nfaPPA group.

Table 2 shows the BAs with rCBF in each group. In lvPPA, svPPA, and nfaPPA patients, rCBF in BA39 right (rt.) was 0.877 ± 0.156, 1.088 ± 0.0659, and 1.101 ± 0.0377, respectively (lvPPA vs. svPPA vs. nfaPPA; *p* = 0.011); in BA39 left (lt.) was 0.791 ± 0.101, 0.977 ± 0.0998, and 1.046 ± 0.0847, respectively (lvPPA vs. svPPA vs. nfaPPA; *p* = 0.015); and in BA40 rt. was 0.860 ± 0.0933, 0.979 ± 0.0147, and 1.047 ± 0.0748, respectively (lvPPA vs. svPPA vs. nfaPPA; *p* = 0.0090), showing that there were significant differences among the 3 groups in BA39 rt., BA39 lt., and BA40 rt. (Figure 3).

**TABLE 2:** Comparison of cerebral blood flow in various BAs among the three PPA groups.

	lvPPA (*n* = 7)	svPPA (*n* = 4)	nfaPPA (*n* = 2)	*p*
BA44 rt.	0.947 ± 0.0764	0.956 ± 0.0434	1.034 ± 0.0519	0.28
BA44 lt.	0.881 ± 0.0819	0.843 ± 0.0551	0.893 ± 0.103	0.65
BA45 rt.	0.935 ± 0.119	0.974 ± 0.0676	1.026 ± 0.0045	0.46
BA45 lt.	0.867 ± 0.109	0.864 ± 0.0454	0.801 ± 0.103	0.79
BA20 rt.	0.732 ± 0.121	0.816 ± 0.190	0.886 ± 0.0653	0.26
BA20 lt.	0.677 ± 0.0453	0.678 ± 0.248	0.809 ± 0.0233	0.21
BA21 rt.	0.829 ± 0.147	0.954 ± 0.161	1.051 ± 0.0614	0.12
BA21 lt.	0.754 ± 0.0629	0.793 ± 0.220	0.923 ± 0.0621	0.16
BA38 rt.	0.667 ± 0.108	0.745 ± 0.335	0.695 ± 0.0437	0.92
BA38 lt.	0.627 ± 0.630	0.607 ± 0.347	0.627 ± 0.0837	0.37
BA39 rt.	0.877 ± 0.156	1.088 ± 0.0659	1.101 ± 0.0377	< 0.05
BA39 lt.	0.791 ± 0.101	0.977 ± 0.0998	1.046 ± 0.0847	< 0.05
BA40 rt.	0.860 ± 0.0933	0.979 ± 0.0147	1.047 ± 0.0748	< 0.01
BA40 lt.	0.776 ± 0.0615	0.897 ± 0.101	0.987 ± 0.127	0.053
BA22 rt.	0.917 ± 0.146	1.064 ± 0.0889	1.115 ± 0.0538	0.062
BA22 lt.	0.839 ± 0.135	0.895 ± 0.135	1.001 ± 0.0826	0.16
BA23 + 31 rt.	0.853 ± 0.0428	0.954 ± 0.0934	0.980 ± 0.153	0.073
BA23 + 31 lt.	0.834 ± 0.0300	0.955 ± 0.115	0.946 ± 0.165	0.22

Abbreviations: BA: Brodmann area; lt.: left; rt.: right.

## 4 Discussion

In this study, a large proportion of lvPPA patients (86%) were amyloid positive and had a characteristic AD pattern on SPECT. These findings suggested that SPECT may play a supportive role in differentiating subtypes of PPA.

Anti‐amyloid‐β monoclonal antibodies were recently approved as the first disease‐modifying therapy for AD. It is important to note that antibody therapies are indicated not only for typical amnesic AD but also for lvAD. According to a previous report, of 54 patients with PPA, 11 were diagnosed as having lvAD and were considered to be eligible for lecanemab treatment [6]. Although the patients in this study were collected before the approval of antibody therapies, 5 of 7 lvAD patients potentially met the criteria for lecanemab or donanemab treatment eligibility. One patient, however, appeared ineligible owing to a low MMSE score of 16 points caused primarily by aphasia, despite relatively preserved activities of daily living. If alternative assessment criteria tailored specifically for aphasic patients become available, patients similar to this patient might also become eligible for antibody therapies in the future. These observations suggested that there may be a substantial number of patients who are potentially eligible for antibody therapies. Situations in which patients who are actually eligible for anti‐amyloid‐β treatment but are unable to receive it simply because they are not properly diagnosed must be prevented. In memory clinics, some patients present with language difficulties. It is therefore crucial to accurately diagnose lvAD in these patients so that they are able to receive appropriate antibody therapies.

On the other hand, there was 1 patient in the lvPPA group who was negative for AD biomarkers in our study. At disease onset, the patient presented predominantly with impaired single‐word retrieval and phrase repetition, without clear agrammatism or semantic impairment, and was therefore diagnosed as having lvPPA due to non‐AD pathology because AD biomarkers were negative. However, approximately 2 years after onset, the patient developed progressively worsening frontal lobe–associated behavioural symptoms, including stereotyped and routinized behaviours, impulsive and excessive spending, and a loss of empathy. Because frontal lobe–associated behavioural symptoms became more prominent than language impairment, the patient was ultimately diagnosed as having frontotemporal lobar degeneration (FTLD). A retrospective review of this patient's cerebral perfusion SPECT demonstrated predominant hypoperfusion in the frontal lobe (Figure 2), which represented an atypical pattern for AD and was instead more suggestive of FTLD. This single counterexample suggests that SPECT may be a useful supportive tool for differentiating patients with lvAD from those with other PPA variants.

One of the 2 patients with nfaPPA showed a decreased CSF Aβ42/40 ratio of 0.067 (A+), whereas amyloid PET was negative with a Centiloid value of approximately 10 (A−), resulting in a discrepancy between the 2 amyloid biomarkers. This result suggests that the nfaPPA group may include patients with very early stage AD, because it has been reported that if no AD pathology is detected on amyloid PET but AD pathology is detected by CSF analysis, the patient may be in a very early stage of AD [26]. In addition, because the core feature of lvPPA, namely, repetition impairment, is sometimes seen in nfaPPA patients [27], it may not be possible to distinguish patients with lvPPA from those with nfaPPA in the early stages of disease onset. In this patient, SLTA revealed clear agrammatism and apraxia of speech, whereas neither single‐word retrieval nor phrase repetition impairment was observed. Therefore, based on the core features of each PPA subtype, the patient was confidently diagnosed as having nfaPPA. However, in the future, if single‐word retrieval and phrase repetition impairment emerge over time and the aphasia phenotype shifts from nfaPPA to lvPPA, the patients' amyloid PET may become positive. Therefore, it is important to perform neuropsychological tests including SLTA, to monitor the clinical course of the symptoms. Although the patient met the guidelines‐based eligibility criteria for anti‐amyloid‐β antibody therapy based on CSF Aβ positivity (A+), careful consideration is required regarding treatment initiation in patients demonstrating a discrepancy between amyloid biomarkers.

Although the use of anti‐Aβ antibody treatments generally requires the confirmation of Aβ pathology in the patient by CSF analysis or amyloid PET, it is more favourable to perform SPECT, which is more accessible and less invasive, as the initial testing method. CSF analysis requires a lumbar puncture, which is invasive and has various risks, including headache [28], nerve root pain, infection, and brain herniation. In addition, patients taking oral antithrombotic medications or those with coagulation abnormalities are at increased risk of bleeding [29]. Furthermore, amyloid PET requires expensive, specialised equipment that is typically available only in large medical centers. Therefore, SPECT, which is less invasive and widely available in general hospitals, may be considered as a screening tool prior to CSF analysis or amyloid PET. If findings on SPECT suggest lvAD, further evaluation using CSF analysis or amyloid PET should be actively performed.

This study has several limitations. Firstly, the accuracy of SLTA may be affected by the skill of the individual performing the test. However, in this study, one skilled neuropsychologist performed all SLTA examinations, so there was no interrater variability. Another potential limitation was the small number of PPA patients. As PPA is a rare syndrome, the number of patients was limited to a very small number as they were collected from only a single center. The small overall number of PPA patients in the present study may have affected the results of the statistical analyses of the comparisons between the three groups. This may be why the areas of cerebral hypoperfusion on SPECT did not differ significantly in all regions, as has been hypothesized previously. The results of statistical analyses with a larger number of patients collected from several institutions should be performed in the future.

In conclusion, our results from this study suggest that SPECT may play a supportive role in differentiating lvAD from PPA. In the current clinical era of anti‐amyloid‐β monoclonal antibody therapy, accurate identification of lvAD among patients with language‐predominant symptoms is increasingly important. A stepwise diagnostic approach starting with clinical language assessment and SPECT, followed by confirmatory biomarker testing when appropriate, may therefore be clinically meaningful. Larger studies are needed to validate these findings.

## Funding

The authors have nothing to report.

## Conflicts of Interest

The authors declare no conflicts of interest.

## Data Availability

The data that support the findings of this study are available from the corresponding author upon reasonable request.
